# The Gut Microbiota Contributes to Systemic Responses and Liver Injury in Gut-Derived Sepsis

**DOI:** 10.3390/microorganisms11071741

**Published:** 2023-07-03

**Authors:** Meiqi Zhao, Jiajia Ma, Huiru Liu, Ying Luo, Huiting Deng, Dandan Wang, Fengmei Wang, Peng Zhang

**Affiliations:** 1 School of Medicine, Nankai University, Tianjin 300071, China; meiqizhao@mail.nankai.edu.cn; 2 Department of Gastroenterology and Hepatology, Nankai University Affiliated Third Central Hospital, Tianjin 300072, China; 3 The Third Central Clinical College of Tianjin Medical University, Tianjin 300070, China; 4 Tianjin Key Laboratory of Extracorporeal Life Support for Critical Diseases, Institute of Hepatobiliary Disease, Nankai University Affiliated Third Central Hospital, Tianjin 300072, China; 5 Life and Health Intelligent Research Institute, Tianjin University of Technology, Tianjin 300387, China

**Keywords:** antibiotics, gut microbiota, inflammation, liver injury, sepsis

## Abstract

The gut microbiota, as a major source of opportunistic pathogens, poses a great threat to systemic infection, whereas the role of the gut microbiota in sepsis is underestimated. Here, we aimed to explore the effects of different gut microbiota patterns (namely, enterotypes) in cecal ligation and puncture (CLP)-induced murine sepsis. To achieve this purpose, we built four kinds of enterotypes by exposing mice to different types of antibiotics (azithromycin, amoxicillin, metronidazole, and levofloxacin). The results showed that antibiotic exposure induced different enterotypes, which, in turn, led to varying levels of systemic inflammation in septic mice, with amoxicillin-associated enterotypes exhibiting the most severe inflammation, followed by metronidazole, azithromycin, and levofloxacin. Specifically, the amoxicillin-associated enterotype was characterized by an abundance of intestinal opportunistic pathogens, including Enterobacteriaceae, Sutterellaceae, and Morganellaceae. This enterotype played a significant role in promoting the pathogenic potential of the gut microbiota, ultimately contributing to the development of severe systemic inflammation. Furthermore, the amoxicillin-associated enterotype exaggerated the sepsis-related liver injury, as evidenced by higher levels of alanine aminotransferase, aspartate transaminase, and hepatic malondialdehyde. The results of the RNA sequencing and the fecal suspension intraperitoneal injection sepsis model indicated that the amoxicillin-associated enterotype provoked acute hepatic immune responses and led to more significant metabolic compensation in the event of sepsis. Collectively, we concluded that the gut microbiota was one crucial factor for heterogeneity in sepsis, where the modulated gut microbiota likely prevented or reduced the serious consequences of sepsis, at least in gut-derived sepsis.

## 1. Introduction

Sepsis is a life-threatening disease, which is often complicated by inflammation and aberrant immune responses, challenging global health [1]. In 2017, the global incident cases of sepsis were estimated to be 48.9 million, including 11 million sepsis-related deaths, which accounted for almost 20 percent of all deaths worldwide [2]. In addition to vulnerable populations (such as older persons, pregnant women, and neonates), anyone influenced by bacterial infection, or serious non-communicable diseases can progress to varying degrees of sepsis [3]. The variation in disease levels can be attributed to the heterogeneity in sepsis, which arises from clinical factors, individual physical characteristics, and the progression of the disease [4]. However, the significance of the gut microbiota as the primary cause of bacterial infection is frequently overlooked. Comprehensively understanding the heterogeneity of sepsis is of great importance to improving targeted treatments of sepsis [5]; thus, deciphering the influence of the gut microbiota on sepsis becomes an indispensable part of the fight against sepsis.

The gut microbiota, comprising of bacteria, archaea, or fungi that inhabit the intestines, serves a crucial function in regulating gut barrier function [6], maintaining intestinal and systematic immune response [7], and protecting the human body from the attack of gut-origin pathogens [8]. Prior studies indicated that gut dysbiosis could induce immune responses [9] and elevate gut permeability [10], which dramatically increases sepsis susceptibility. Recently, the largest contributor to sepsis cases was ascertained as diarrheal diseases, and patients with cirrhosis, meningitis, diabetes, and malnutrition are also at high risk for sepsis [2,11]; therefore, the gut microbiota is likely to be the chief culprit. In clinical practice, oral antibiotic therapies or antibiotic prophylaxis inevitably disorders gut microbiota homeostasis, evoking the overgrowth of specific gut bacteria and enrichment of antimicrobial resistance [12,13], which can rapidly lead to the deteriorating clinical conditions of sepsis, or even death [14]. The crosstalk between sepsis and the gut microbiota was further addressed, where Group B *streptococcus* and *Escherichia coli* were identified as the major causes of sepsis [15,16], and microbiota-derived short-chain fatty acid (acetate, propionate, and butyrate) and gut-derived lipopolysaccharide (LPS) were confirmed to be associated with sepsis pathogenesis [17,18].

The sepsis-related infection can spread quickly throughout the body and worsen without timely treatment, causing septic shock, multiple organ failure (such as the lungs, kidneys, and liver), and even death [19]. Notably, the liver also plays a crucial role in regulating the immune defense against systemic infections via bacterial clearance, the production of acute-phase proteins or cytokines, and the adaptation of hepatic biosynthesis and metabolism to inflammation [20,21]. Clinical evidence demonstrates that sepsis could cause liver damage, leading to hypoxic hepatitis, sepsis-induced cholestasis, and secondary sclerosing cholangitis [22,23]. Additionally, sepsis-related liver dysfunction is highly relevant to the prognostic effects of sepsis, where the key markers include serum bilirubin levels and the serum activities of aspartate aminotransferase (AST) and alanine aminotransferase (ALT) [24,25,26]. Furthermore, liver dysfunction and immune responses are recognized as the main drivers of the progression of liver injury during sepsis; thus, a comprehensive understanding of liver responses to sepsis is urgently needed.

To better depict the role of gut microbiota in the sepsis heterogeneity, the current study employed four kinds of commonly used oral antibiotics (azithromycin, amoxicillin, metronidazole, and levofloxacin) to generate distinct gut microbiota, or enterotypes, in mice. The concentration of antibiotics was carefully selected based on the considerations of absorption and bioavailability in the murine intestine, as well as the human equivalent dose [27,28]. Then, we explored the effects of these enterotypes on systemic inflammation and liver injury in mice with sepsis induced by cecal ligation and puncture (CLP). These findings support the pivotal role of the gut microbiota in the advancement of sepsis and provide evidence that the sepsis sensitivity was likely associated with the gut microbiota, which suggests that targeting the gut microbiota might be a potential therapeutic strategy against sepsis.

## 2. Materials and Methods

### 2.1. Animal Model

The sixty male C57BL/6 mice (6–7 weeks) used in this experiment were purchased from Beijing Hua Fukang Co., Ltd. (Beijing, China). All mice were maintained in a temperature-controlled (temperature at 22 ± 2 °C, humidity at 50 ± 15%) and specific pathogen-free (SPF) conditions on a 12 h day/night cycle. Furthermore, the mice were given ad libitum access to sterile food and water. For the CLP model, the co-housed mice were randomly assigned to 6 experimental groups (10 mice per group) before the experiment, which were defined as the sham (no CLP + water), water (CLP + water), azithromycin (CLP + azithromycin), amoxicillin (CLP + amoxicillin), metronidazole (CLP + metronidazole), and levofloxacin (CLP + levofloxacin) groups. During the experiment, the mice were treated with water (200 μL per mice in the sham and water groups) or antibiotics separately via oral gavage for 3 days, where the commonly used oral antibiotics were azithromycin (25 mg/kg body weight/day), amoxicillin (25 mg/kg body weight/day), metronidazole (50 mg/kg body weight/day), and levofloxacin (50 mg/kg body weight/day). Thereafter, a moderate cecal ligation and puncture (CLP) was performed as previously described [29], and the mice were sacrificed via cervical dislocation 24 h after the CLP. Liver tissues were collected and weighed under sterile conditions, and blood was collected and centrifuged (3000× *g*, 4 °C for 15 min) to obtain serum for further analysis. To determine the role of the gut microbiota in sepsis, we performed the following analyses: RNA sequencing, reverse transcription quantitative PCR (RT-qPCR), 16S ribosomal RNA (rRNA), biochemistry experiments, total bacterial load in feces (Supplementary Text S1), and histopathology of liver tissues (Supplementary Text S2). To exclude the influence of the antibiotic treatment on mice, the antibiotics azithromycin, amoxicillin, metronidazole, and levofloxacin were administrated separately to mice as indicated above (6 mice per group), and the blood and liver were collected and analyzed two days after the last gavage.

### 2.2. Fecal Suspension Intraperitoneal Injection Experiments

To verify the high-risk potential of the enterotype induced by amoxicillin exposure, a fecal suspension (FS) intraperitoneal injection (IP) model was adopted as previously described [30]. Briefly, the fresh feces that were collected from the mice of the water (without antibiotic treatment) and amoxicillin groups just before the CLP surgery were prepared with a sterile saline solution, and the fecal suspension (20 mg/mL) was used within 2 h. Before the experiments, the co-housed mice were randomly assigned to 3 groups (6 mice per group), which were defined as the control (IP: sterile saline solution), water–feces (IP: fecal suspension from water group), and amoxicillin–feces (IP: fecal suspension from amoxicillin group) groups. A total of 1 mL fecal suspensions (1 × 10^8^ copy number/mL) were intraperitoneally administered to each mouse, while a sterile saline solution was used as the vehicle control. Twenty hours later, the mice were sacrificed via cervical dislocation, and the blood and liver tissues were collected for further analysis.

### 2.3. RNA Sequencing and RT-qPCR

RNA sequencing was employed to profile the transcriptional landscape of the liver in polymicrobial sepsis, and the experiments were implemented at the Beijing Novogene Bioinformatics Technology Co., Ltd. (Beijing, China). Specifically, the libraries were built with a purified RNA sample (3 μg) using the NEBNext Ultra II RNA Library Prep Kit for Illumina (NEB, Ipswich, MA, USA) according to the manufacturer’s instructions, and the NovaSeq 6000 platform was employed to generate raw data (150 bp paired-end reads). For the sham, water, and amoxicillin groups, averages of 37.8 Mb, 41.08 Mb, and 42.74 Mb high-quality reads, respectively, were generated using RNA-seq. HISAT2 was used to align the sequencing reads to the mouse reference sequence (UCSC/GRCm39) [31], StringTie was used to assemble and quantitate RNA-Seq alignments [32], and DESeq was employed to screen for differentially expressed genes (DEGs) with the criteria of |log_2_ foldchange (FC)| > 1 and false discovery rate (FDR) < 0.01, and therefore, FDR was obtained via adjusting the *p*-values using the Benjamini–Hochberg method [33]. In addition, DEGs were annotated based on the Kyoto Encyclopedia of Genes and Genomes (KEGG) database, and the entire set of gene expression data was interpreted via Gene Set Enrichment Analysis (GSEA) [34], where pathways with FDR < 0.05 were considered statistically significant. RT–qPCR was used to verify the partial DEGs among the sham, water, and amoxicillin groups; the primers used are listed in Table S1.

### 2.4. 16S rRNA Sequencing

To analyze the alterations to the fecal gut microbiota, fresh feces were collected before the CLP surgery and stored at −80 °C before use. Total bacterial DNA from fecal samples was prepared using the DNeasy PowerSoil Kit (Qiagen, Hilden, Germany) in line with the manufacturer’s instructions, and the quality of the prepared DNA was verified via gel electrophoresis. PCR was performed by targeting the V3–V4 region of the 16S rRNA. After purification, the high-quantity samples were sequenced using the Illumina Novaseq 6000 platform (2 × 250 bp, San Diego, CA, USA).

The raw sequence data were processed following the QIIME 2 pipeline [35], and the operational taxonomic units (OTUs) and the percentage of relative abundance were computed and normalized. The alpha diversity based on the OTUs was calculated for the groups, and the principal coordinates analysis (PCoA) based on Bray–Curtis distances was used to determine the beta diversity of the gut microbiota. PICRUSt2 was used to infer the metagenomes of the gut microbiota [36], and BugBase was employed to predict the pathogenic potential of the gut microbiota [37]. These data were analyzed with R version 4.2.1 and GraphPad Prism (version 8.0).

### 2.5. Biochemistry Experiments

The levels of ALT, AST, and total cholesterol (CHO) in the serum were determined using the Beckman Coulter AU5800 chemistry system. The cytokine levels of TNF-α and IL-6 in the serum were examined with ELISA kits (Solarbio, Beijing, China) according to the manufacturer’s protocols. The level of hepatic malondialdehyde (MDA), which is a biomarker of lipid peroxidation, was assessed by using the MDA content detection kit (Solarbio, China).

### 2.6. Statistical Analysis

GraphPad Prism (version 8.0) and R (version 4.2.1) were used for the data analysis and graphic representation, and data are shown as mean ± standard error (SD). Significance was tested using the unpaired two-tailed Student’s *t*-test for two groups, and one-way ANOVA followed by Tukey-test with adjusted *p*-values was employed for multiple comparisons. The *p*-values were adjusted using the Benjamini–Hochberg method where appropriate. Data were considered statistically significant between groups according to * *p* < 0.05 and ** *p* < 0.01.

## 3. Results

### 3.1. Distinct Gut Microbiota Influenced the Systemic Injury in Sepsis Mice

**Figure 1:**
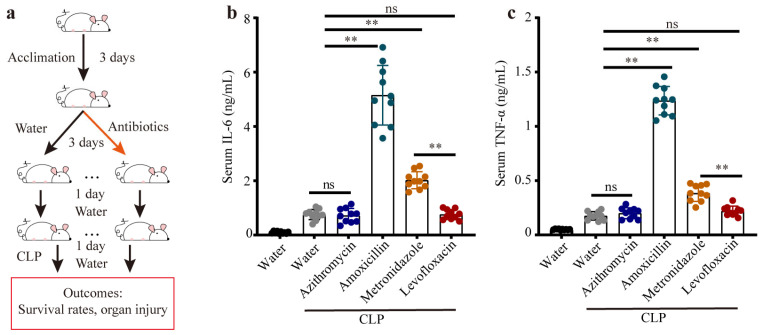
Previous antibiotic exposure elevated systemic inflammation in CLP mice. (**a**) The experimental design: mice received different antibiotics for 3 days, namely, azithromycin, amoxicillin, metronidazole, and levofloxacin, after which moderate CLP was performed, and the mice were sacrificed 24 h after the CLP. The serum levels of IL-6 (**b**) and TNF-α (**c**). ns: no significant difference, ** *p* < 0.01.

To mimic different gut microbiome compositions, we exposed mice to commonly used antibiotics (namely, azithromycin, amoxicillin, metronidazole, and levofloxacin) separately for three days. Forty-eight hours after the last intervention of antibiotics (to avoid the influence of antibiotics in the mice), the experimental model of sepsis was implemented on these mice (Figure 1a). Then, we explored the inflammation responses during sepsis, where the serum levels of the inflammatory markers (IL-6 and TNF-α) were determined. We observed that the mice pretreated with amoxicillin showed exaggerated systemic inflammation, which was characterized by remarkably increased levels of serum IL-6 and TNF-α, followed by the metronidazole-treated mice (Figure 1b,c). Moreover, the mice pretreated with azithromycin or levofloxacin showed no significant difference in inflammation responses compared with the CLP mice that received water (Figure 1b,c). Collectively, mice with CLP-induced sepsis exhibited different sensitivities to the distinct gut microbiota, indicating that the gut microbiota likely played a central role in the progression of polymicrobial sepsis.

### 3.2. Previous Antibiotic Exposure Caused Different Enterotypes in Mice

**Figure 2:**
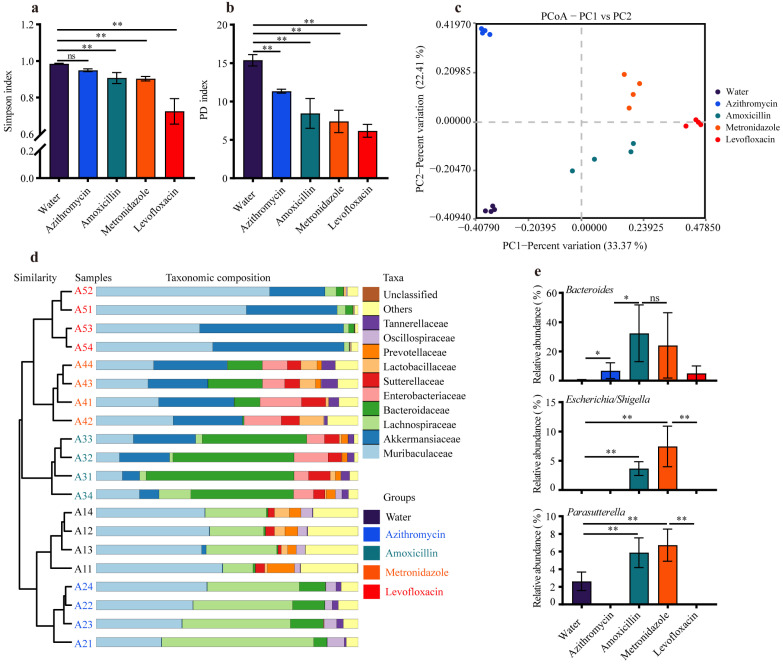
Antibiotic exposure induced disparate enterotypes in mice. The alpha diversity analysis, as measured using the (**a**) Simpson index and (**b**) Faith’s PD based on the OTU table. (**c**) Principal coordinates analysis (PCoA) plot generated from the Bray–Curtis distance matrix. (**d**) The average relative abundance of the gut microbiota at the family level and the cluster was based on the Bray–Curtis distances. Only the 10 most abundant families are shown. (**e**) The average relative abundances of bacterial species, including *Bacteroides*, *Escherichia*/*Shigella*, and *Parasutterella*. ns: no significant difference, * *p* < 0.05, ** *p* < 0.01.

To characterize the gut microbiota alterations that caused the heterogeneity in sepsis, we performed a gut microbiota analysis. The 16S rRNA sequencing analysis revealed that previous antibiotic exposure considerably reduced the α-diversity compared with the water group, as represented by a reduced Simpson index and phylogenetic diversity (PD) index (Figure 2a,b). Contrary to the results of inflammation responses, the mice pretreated with levofloxacin showed the most dramatic changes in gut microbiota diversity (Figure 2a–c), and the low pathogenic potential of the specific enterotype was likely associated with the dominant flora. Additionally, the results of the PCoA based on the Bray–Curtis distance demonstrated that clusters of gut microbiota among the water- and antibiotics-treated mice were completely separated (Figure 2c), indicating gut microbiota compositions exhibited remarkable differences between different groups.

In agreement with the reduced diversity, previous antibiotic exposure remarkably reduced the variety in the gut microbiota at the levels of family and genus (Figure S1), whereas no significant difference in total bacterial load of fecal samples was found between the water- and antibiotic-treated mice (Figure S2). The taxonomic composition analysis also revealed that antibiotic exposure significantly changed the bacterial abundance at both the phylum and family levels (Figure 2d and Figure S3). To further identify which bacterial taxa were special among these water- and antibiotic-treated groups, we implemented LEFSe and found distinctive genera in each group (Figure S4). The phylum Bacteroidota (Muribaculaceae) was the predominant phyla of all the groups (>40%), and the administration of amoxicillin or metronidazole considerably promoted the growth of Enterobacteriaceae (*Escherichia*/*Shigella*, *proteus*), Sutterellaceae (*Parasutterella*), and Morganellaceae (Figure 2d,e and Table S2), which are the main sources of bacterial infections in humans [38,39]. Additionally, the administration of azithromycin significantly elevated the relative abundance of Lachnospiraceae (*Lachnospiraceae* NK4A136 group and *Lachnospiraceae* UCG 006) and Oscillospiraceae, and the administration of levofloxacin increased the relative abundance of Akkermansiaceae (*Akkermansia*) and Muribaculaceae (Figure 2d and Table S2). These intestinal bacteria, particularly Lachnospiraceae, Akkermansiaceae, and Muribaculaceae, are known as commensal bacteria that reside in the gastrointestinal tract to supply the host with essential nutrients [40,41], which was likely responsible for the lower systemic inflammation in these sepsis mice pretreated with azithromycin or levofloxacin.

### 3.3. Previous Antibiotic Exposure Disordered the Function of Gut Microbiota in Mice

**Figure 3:**
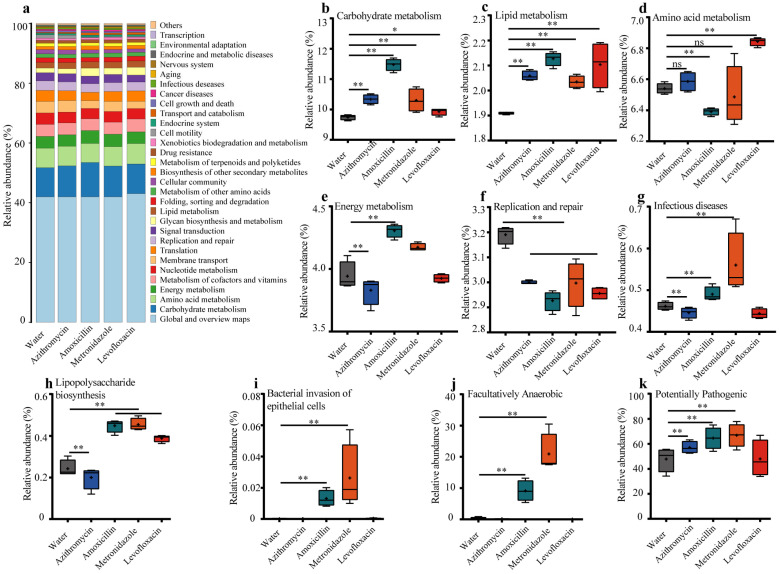
Antibiotic exposure disordered the function of the gut microbiota in CLP mice. (**a**) The relative abundances of KEGG pathways at level 2, as predicted using PICRUSt2. The relative abundances of the KEGG pathways at level 3: (**b**) carbohydrate metabolism, (**c**) lipid metabolism, (**d**) amino acid metabolism, (**e**) energy metabolism, (**f**) replication and repair, (**g**) infectious diseases, (**h**) lipopolysaccharide biosynthesis, (**i**) bacterial invasion of epithelial cells, and (**j**) facultatively anaerobic. (**k**) The potentially pathogenic level of the gut microbiota as predicted using Bugbase. ns: no significant difference, * *p* < 0.05, ** *p* < 0.01.

Different enterotypes confer disparate functions to the gut microbiota, which participate in regulating nutrient metabolism and immune homeostasis in the host [42]. To understand the functional implications of the observed taxonomic alterations, we inferred metagenomes using PICRUSt2. The relative abundances of KEGG pathways are shown in Figure 3 (Table S3), which illustrates that antibiotic administrations considerably altered the functions of the gut microbiota, including carbohydrate metabolism, lipid metabolism, amino acid metabolism, energy metabolism, and the replication and repair (Figure 3a–f). Additionally, the administration of amoxicillin or metronidazole notably increased the genes involved in infectious diseases of the gut microbiota, as well as these genes associated with lipopolysaccharide biosynthesis and bacterial invasion of epithelial cells (Figure 3g–j). Specifically, we observed that the treatment of amoxicillin or metronidazole significantly increased the relative abundances of facultatively anaerobic bacteria, which was dominated by Enterobacteriaceae (Figure 3k, Table S4). Collectively, these results indicate the higher pathogenic potential of the specific enterotype induced by amoxicillin, which was further confirmed by the BugBase analysis (Figure 3i). In this context, we concluded that the compositional and functional alterations of the gut microbiota induced by amoxicillin were likely associated with higher systemic inflammation in the CLP-induced-sepsis mice.

### 3.4. The Polymicrobial Sepsis Impaired the Liver Function in Mice

**Figure 4:**
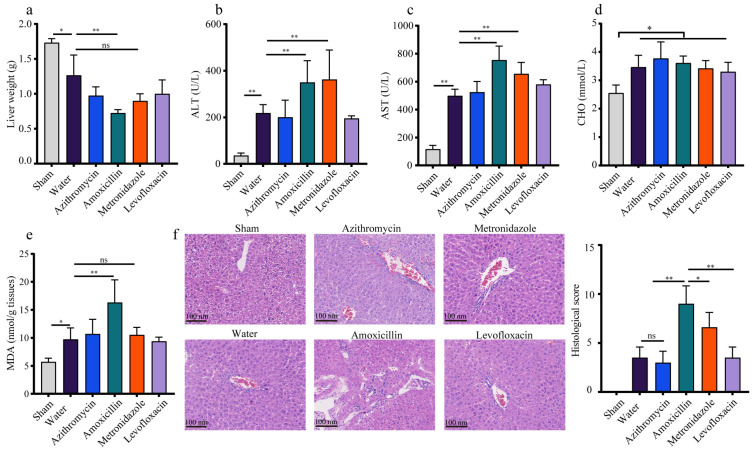
Exposure to different antibiotics caused liver injuries to different extents in CLP mice. (**a**) The liver weight. The enzymatic activities of (**b**) ALT and (**c**) AST in serum. (**d**) The serum level of CHO. (**e**) The MDA level in liver tissues. (**f**) H&E staining and the histological score of the liver. ns: no significant difference, * *p* < 0.05, ** *p* < 0.01.

To dissect the potential threats of polymicrobial sepsis to organs, we chose the liver, which is recognized as the guardian, modifier, and target of sepsis in the host, to determine its pathophysiological changes via biochemical experiments. The results suggested that compared with the sham-operated mice, all the sepsis mice (with or without antibiotic treatment) exhibited obvious liver injury, as represented by a lower liver weight, elevated serum ALT and AST activity, and increased serum CHO level and hepatic MDA concentrations (*p* < 0.05, Figure 4a–e). Additionally, we did not observe any significant difference for a single antibiotic treatment on the aforesaid liver indexes in mice (*p* > 0.05, Figure S5a–g); thus, the role of these antibiotics in the sepsis model was ignored in this study. Taken together, these results suggest that the CLP surgery dramatically disordered the liver function in mice.

**Figure 5:**
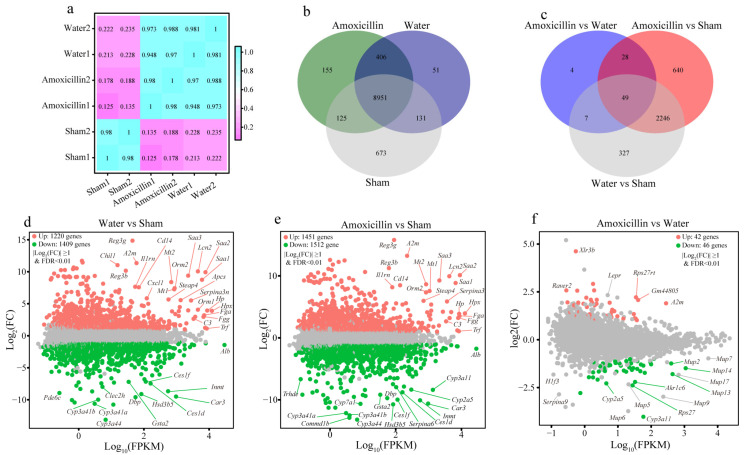
The CLP surgery caused transcriptional alterations in the liver tissues. (**a**) Correlation heatmap of gene expressions between the sham, water, and amoxicillin groups, where Pearson’s correlation coefficient was used to evaluate the correlation of biological repetition for the RNA-sequencing data. (**b**) The co-expressed genes among the sham, water, and amoxicillin groups from the RNA-sequencing data. (**c**) The co-shared DEGs among the sham, water, and amoxicillin groups. MA plots are used to represent the log_2_(FC) versus log_10_(FPKM) (mean expression) between (**d**) the water and sham groups, (**e**) the amoxicillin and sham groups, and (**f**) the amoxicillin and water groups. The screening criteria of DEGs were |log2(FC)| ≥ 1 and FDR < 0.01, FC: fold change, FPKM: fragments per kilobase million, FDR: false discovery rate, up: upregulated genes, down: downregulated genes.

We applied RNA sequencing to profile the whole transcriptional landscape of the liver in polymicrobial sepsis and observed that CLP surgery obviously disordered the hepatic transcription in the mice of the water (CLP + water) and amoxicillin (CLP + amoxicillin) groups (Figure 5a–c). In comparison with the sham-operated mice, the CLP mice that received water showed 2629 differentially expressed genes (DEGs) in liver tissues, including 1220 upregulated genes and 1409 downregulated genes (Figure 5d and Table S5). These upregulated DEGs were enriched in 22 KEGG pathways and showed a statistical difference (*p* < 0.05, Figure S6a), which was mainly associated with human diseases, such as the pathways of shigellosis, salmonella infection, hepatitis C, and Epstein–Barr virus infection. Furthermore, the pathways of NOD-like receptor signaling, IL-17 signaling, TNF signaling, and NF-kappa B signaling were enriched with the upregulated DEGs, as well as the pathways of complement and coagulation cascades (Figure S6a), which indicated that CLP surgery significantly activated the murine immune responses. In contrast, these 48 downregulated KEGG pathways were largely involved in liver metabolism, including amino acid metabolism, fatty acid metabolism, carbohydrate metabolism, and xenobiotics biodegradation and metabolism (*p* < 0.05, Figure S6b). Specifically, the pathways of peroxisome, bile secretion, and metabolism associated with P450 were also significantly enriched with these downregulated DEGs, suggesting that CLP surgery obviously impaired the liver functions of metabolism, detoxification, and bile secretion (*p* < 0.05, Figure S6b). The GESA analysis also confirmed that in liver tissues, the immune responses were activated, and the liver function of metabolism was repressed during polymicrobial sepsis (*p* < 0.05, Table S6).

### 3.5. Previous Administration of Amoxicillin Exaggerated the Sepsis-Induced Liver Injury in Mice

The biochemical experiments demonstrated that these sepsis mice pretreated with amoxicillin received the most severe liver injuries among all the groups, and were characterized by the lowest liver weight, higher levels of serum ALT and AST, and a higher hepatic MDA level (Figure 4a–e), which was further verified by the histological analysis (Figure 4f). Using RNA sequencing, we deciphered the influence of the amoxicillin-associated enterotype on sepsis-related liver injury in detail. Compared with the sham-operated mice, the CLP mice pretreated with amoxicillin exhibited remarkably disordered hepatic transcripts, as characterized by 2963 DEGs, including 1451 upregulated genes and 1512 downregulated genes (Figure 5e and Table S7). The KEGG pathway analysis demonstrated that these upregulated genes were mainly enriched in pathways of human diseases associated with infection and immune responses, and these downregulated genes were primarily involved in liver metabolic processes (Figure S7).

**Figure 6:**
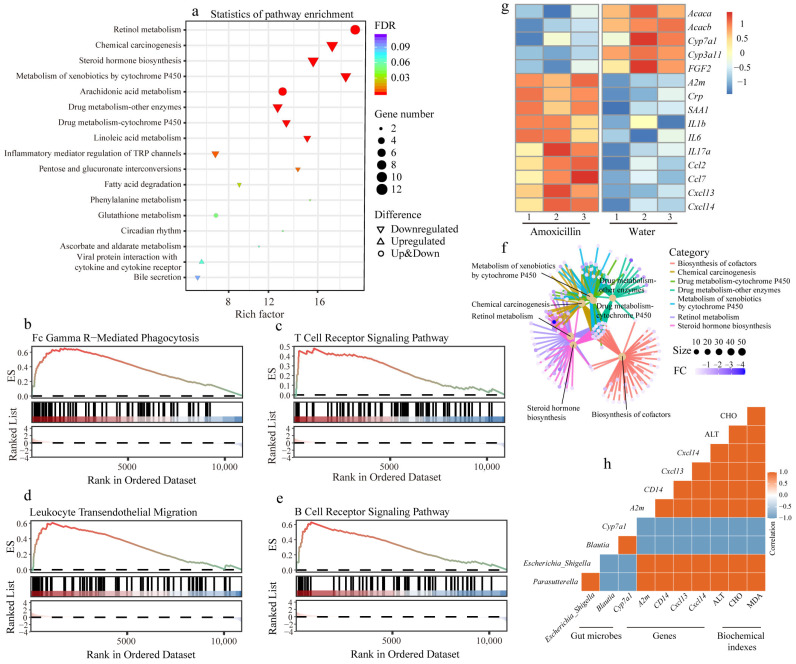
The specific enterotype induced by amoxicillin treatment exaggerated liver injury in CLP mice. (**a**) The top 17 enriched KEGG pathways based on DEGs between the amoxicillin and water groups. The pathways associated with immune responses enriched by GSEA based on the whole transcripts, including (**b**) Fc gamma r-mediated phagocytosis, (**c**) T cell receptor signaling pathway, (**d**) leukocyte transendothelial migration, and (**e**) B cell receptor signaling pathway. (**f**) The cnet plot between the KEGG pathways and associated genes. (**g**) Heatmap of the gene expressions analyzed using RT-qPCR, including genes *Acaca*, *Acacb*, *Cyp7a1*, *Cyp3a11*, *FGF2*, *A2m*, *Crp, SAA1*, *IL1b*, *IL6*, *IL17a*, *Ccl2*, *Ccl7*, *Cxcl13*, and *Cxcl14*. (**h**) The Pearson correlation analysis among gut microbes (*Parasutterella*, *Escherichia*/*Shigella*, and *Blautia*), several marker genes (*Cyp7a1*, *A2m*, *CD14*, *Cxcl13*, and *Cxcl14*), and biochemical indexes of the liver (ALT, CHO, and MDA). FC: fold change, FDR: false discovery rate, ES: enrichment score.

The differential expression analysis between the water (CLP + water) and amoxicillin (CLP + amoxicillin) groups identified 88 DEGs, including 42 upregulated genes and 46 downregulated genes (Figure 5f). These DEGs were mainly enriched in 10 downregulated pathways, such as chemical carcinogenesis, steroid hormone biosynthesis, metabolism of xenobiotics by cytochrome P450, inflammatory mediator regulation of TRP channels, pentose and glucuronate interconversions, and fatty acid degradation (Figure 6a). In combination with GSEA (Table S8), we found that these downregulated pathways fell further in the sepsis mice pretreated with amoxicillin, which suggested the amoxicillin-associated enterotype likely raised the number of seriously disordered transcripts in the liver of sepsis mice. Additionally, the GESA also identified five KEGG pathways associated with hepatic immune responses, namely, Fc gamma R-mediated phagocytosis, leukocyte transendothelial migration, T cell receptor signaling pathway, B cell receptor signaling pathway, and Fc epsilon RI signaling pathway (Figure 6b–e and Table S8), suggesting that the immune responses were stronger in the amoxicillin-treated sepsis mice. In this context, the liver functions of metabolism were more vulnerable and impaired, as characterized by the repressed pathways of primary bile acid biosynthesis, carbon metabolism, ascorbate and aldarate metabolisms, tryptophan metabolism, etc. (Table S8). Furthermore, the pathway and gene interaction network displayed a close association between these significantly changed liver functions (Figure 6f). Moreover, we corroborated the results of the repressed liver metabolic processes and enhanced the immune response in sepsis mice via RT-qPCR targeting the marker genes, such as *Acaca*, *Acacb*, *Cyp7a1*, *FGF2*, and *A2m* (Figure 6g). Furthermore, the correlation analysis demonstrated that the enriched gut bacteria, such as *Escherichia*/*Shigella* and *Parasutterella*, were significantly positively correlated with the expression of genes related to immune response and the liver function indexes and negatively correlated with the expression of genes associated with liver metabolic processes (Figure 6h), which showed that the amoxicillin-associated enterotype exaggerated the liver injury in sepsis mice.

### 3.6. The Enterotypes Enriched in Pathogenic Microorganisms Aggravated Systemic Inflammation and Liver Injury in Mice

**Figure 7:**
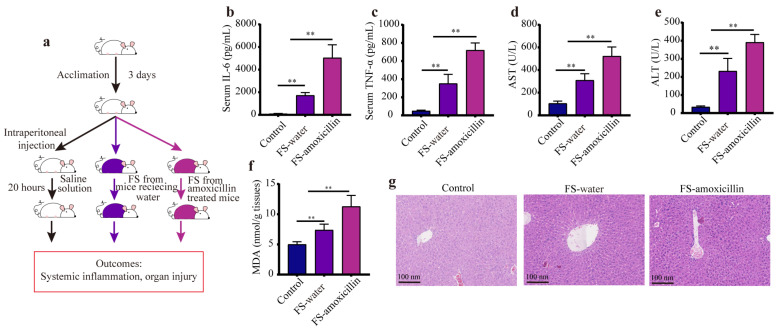
The high-risk potential of amoxicillin-associated enterotypes was confirmed via a fecal suspension intraperitoneal injection model. (**a**) The experimental design: mice were intraperitoneally administered with a different fecal suspension after 3 days of acclimation, including saline solution as the vehicle control, fecal suspension of sham mice (without antibiotic treatment), and fecal suspension (FS) of amoxicillin-treated mice, and mice were sacrificed 20 h after the fecal suspension intraperitoneal injection. The serum levels of IL-6 (**b**) and TNF-α (**c**). The enzymatic activities of (**d**) AST and (**e**) ALT in the serum. (**f**) The MDA level in liver tissues. (**g**) Representative images of H&E staining in the liver. FS: fecal suspension, ** *p* < 0.01.

To further confirm the high-risk potential of the enterotypes (associated with amoxicillin) enriched in pathogenic microorganisms, we built another sepsis model (fecal suspension intraperitoneal injection model) in mice, as shown in Figure 7a. As expected, intraperitoneal administration of fecal suspensions (FS) to these mice resulted in notable inflammation responses, as demonstrated by the significantly increased serum concentrations of IL-6 and TNF-α compared with the mice treated with saline solution (Figure 7b,c). Specifically, the level of systemic inflammation in the mice of the FS–amoxicillin group was remarkably higher than that in the FS–water group, suggesting that fecal suspension associated with amoxicillin induced more severe inflammation responses in mice. Following the intraperitoneal injection of fecal suspension, liver function was significantly impaired, as evidenced by the increased serum levels of AST and ALT in FS–water and FS–amoxicillin mice compared with the control mice. Results from the hepatic MDA concentrations and H&E staining images also revealed that the gut microbiome associated with amoxicillin caused more serious liver injury than that of the enterotypes from the mice without treatment. Taken together, all these findings verified that the gut microbiome enriched in pathogenic microorganisms aggravated systemic inflammation and liver injury in the sepsis mice.

## 4. Discussion

Deciphering the factors for the heterogeneity in sepsis has critical therapeutic implications in the clinic. Prior studies showed us that gut homeostasis is of great importance for sepsis, especially involving gut microbiota and gut permeability [43,44]. However, the influences of different gut microbiota compositions on the outcomes of sepsis patients were still unclear, where the gut microbiota composition represents one of the greatest factors that affect the onset and progression of sepsis.

This study combined high-throughput sequencing and a CLP-induced polymicrobial sepsis model to systematically investigate the influence of disparate enterotypes on sepsis. We observed that the enterotype generated by the amoxicillin treatment caused a more severe systemic inflammation response. Additionally, the pretreatment of amoxicillin considerably disordered the liver function, as characterized by the activated immune responses, and repressed liver metabolic processes, which was likely associated with the altered gut microbiota compositions.

Several studies recognized that the treatment of antibiotics would reshape gut microbiota compositions over a certain period, and the difference in bacterial alteration is largely dependent on the antibiotic types [45,46]. Coupled with frequent exposure in daily life, antibiotics emerge as the major factor in forming gut microbiota heterogeneity [47], especially for the commonly used oral antibiotics. In line with prior studies [48,49], the phyla of Bacteroidota and Firmicutes were predominant in the mice in the water and azithromycin groups. The phyla of Verrucomicrobiota and Proteobacteria replaced the niches of Firmicutes that comprised a mass of intestinal commensal microbes in the amoxicillin and metronidazole groups, and the overgrowth of Proteobacteria was shown to be associated with intestinal diseases and systemic infections [50]. Additionally, the analysis of the function of the gut microbiota revealed that the enterotype generated by the pretreatment of amoxicillin posed higher pathogenic potential, which was evidential support for the enhanced inflammation responses of these mice.

The liver constitutes one key line of defense in fighting against invading bacteria and their products in the circulatory system and subsequently inhibiting the spread of bacteria into the body [51]. In the case of sepsis, the acute-phase responses, bacterial clearance, and complement and coagulation cascades are strongly activated in the liver [52,53]. Of which, elevated C-reactive protein (encoded by *Crp*) binds to invading bacterial cells to activate the complement system (encoded by *C3*, *C4b*, *C4bp*, etc.), which promotes phagocytosis by macrophages targeting apoptotic cells or bacteria [54]. The serum amyloid A (encoded by *SAA1/2/3*) takes part in the innate immune response via the recruitment of leukocytes to the site of inflammation [55]. In response to systemic cytokines and alarmins, the stellate cells and Kupffer cells produce many chemokines (such as Ccl2, Ccl6, Cxcl1, Cxcl10, and Cxcl13) to recruit immune cells (neutrophils and monocytes) and promote the hepatocytes to adapt the inflammation status [20]. Moreover, our results reveal that polymicrobial sepsis also considerably impairs the liver metabolic processes, including the major function of hepatic metabolisms associated with P450, fatty acid metabolism, carbon metabolism, and amine acid. In the setting of sepsis, the immune responses associated with recognizing, binding, and clearing the invading bacteria, as well as their products, likely become the top priority of the liver.

In comparison with the single sepsis mice, these sepsis mice pretreated with amoxicillin exhibited markedly impaired metabolic properties associated with P450, fatty acid, steroid hormone biosynthesis, and enhanced immune responses. These results demonstrated that previous exposure to amoxicillin exaggerated the sepsis-induced liver injury in mice, which was largely on account of the gut microbiota alterations. Amoxicillin exposure and its related gut dysbiosis were reported to be associated with intestinal diseases and liver injury, and the overgrowth of Proteobacteria is likely to blame [56,57]. In the current study, the hepatic transcriptional landscape of the azithromycin, metronidazole, and levofloxacin groups was not characterized. Based on the biochemical experiments and histological analysis, we observed that the enterotypes induced by metronidazole seemed to cause higher systemic inflammation (lower than that of the amoxicillin group) in sepsis mice, while the enterotypes of azithromycin- and levofloxacin-treated mice displayed no significant differences in systemic inflammation. Collectively, we concluded that specific enterotypes would lead to disparate outcomes of polymicrobial sepsis in mice; in other words, the gut microbiota was a vital factor for heterogeneity in sepsis, at least in gut-derived sepsis.

The pathobiology-driven understanding of the heterogeneity in the host response to sepsis has become widely accepted [5,58], and co-opting the gut microbiota into this understanding would drive the advance of improving therapeutic strategies for gut-derived sepsis. As a major source of systemic infection, pathogenic intestinal bacteria pose a serious threat to the gastrointestinal tract and even liver tissues via portal veins [59,60]. For patients diagnosed with sepsis or bacteremia, attempts to alleviate gut dysbiosis or enrich the beneficial gut microbes will likely obtain extraordinary therapeutic effects as supplements of traditional treatments. The major approaches to regulate the gut microbiota include a fecal microbiota transplant and supplementing probiotics, prebiotics, or synbiotics by consuming more probiotic-rich foods or drugs [61,62].

## 5. Conclusions

In summary, by inducing different enterotypes with commonly used oral antibiotics, our work suggested the gut microbiota as a vital factor for the heterogeneity in sepsis (especially gut-derived sepsis) and uncovered an underlying mechanism by which gut dysbiosis induced by amoxicillin elevated inflammation responses and exaggerated related liver injury in sepsis mice. These findings expand our knowledge of gut microbiota in gut-derived sepsis and indicate a potential approach for alleviating sepsis-related injury.

## Data Availability

All processed data supporting the conclusions are presented in the main text and Supplementary Materials. The raw data of the RNA sequencing and 16S rRNA sequencing are available in the CNCB-NGDC (https://ngdc.cncb.ac.cn/gsub/, accessed on 24 September 2022) (accession numbers CRA008297 and CRA008281, respectively). All other original data are available and can be shared upon request by contacting the corresponding authors.

## Supplementary Materials

The following supporting information can be downloaded from https://www.mdpi.com/article/10.3390/microorganisms11071741/s1. Ref. [63] is cited in the Supplementary Materials.
