# Supplementary material for: The Gut Microbiota Contributes to Systemic Responses and Liver Injury in Gut-Derived Sepsis
Source: Microorganisms. 2023 Jul 3;11(7):1741. doi: 10.3390/microorganisms11071741 (PMC10383566; doi:10.3390/microorganisms11071741)
Supplement: Supplementary file 1 [file microorganisms-11-01741-s001.zip › microorganisms-2465518-supplementary.pdf]

The Gut Microbiota Contributes to Systemic Responses and Liver Injury in Gut-Derived Sepsis

Meiqi Zhao [1,2], Jiajia Ma [2,3], Huiru Liu [2,3], Ying Luo [4], Huiting Deng [4], Dandan Wang [4], Fengmei Wang [1,2,*] and Peng Zhang [5,*]

1 School of Medicine, Nankai University, Tianjin 300071, China

2 Department of Gastroenterology and Hepatology, Nankai University Affiliated Third Central Hospital, Tianjin 300072, China

3 The Third Central Clinical College of Tianjin Medical University, Tianjin 300070, China

4 Tianjin Key Laboratory of Extracorporeal Life Support for Critical Diseases, Institute of Hepatobiliary Disease, Nankai University Affiliated Third Central Hospital, Tianjin 300072, China

5 Life and Health Intelligent Research Institute, Tianjin University of Technology, Tianjin 300387, China

* Correspondence: wangfengmeitj@126.com (F.W.); zpeng@email.tjut.edu.cn (P.Z.); Tel.: +86-022-84112114 (F.W.); +86-022-60215055 (P.Z.)

The supplementary information includes three appendices. The first appendix contains information about additional methods and protocols. The second appendix provides details on additional figures. The third appendix describes primers sequences and supplementary data in tables.

I. Supplementary Methods

II. Supplementary Figures

III. Supplementary Tables

I Supplemental Methods

Text S1. Assessment of fecal bacterial load by RT-qPCR

Fecal bacterial DNA was prepared by DNeasy PowerSoil Kit (Qiagen) following the manufacturer's instruction. Primers for bacterial community quantification were UniF340 (ACTCCTACGGGAGGCAGCAGT) and UniR514 (ATTACCGCGGCTGCTGGC). The PCR products based on DNA templates of *E. coli* K-12 MG1655 were cloned into a pUCm-T Vector (Sangon Biotech, Shanghai, China). The cloned plasmid was prepared using Plasmid Mini Kit I (Omega, Norcross, GA, USA), and its concentration was measured using Nanophotometer N60. The corresponding plasmid copy number was calculated with the formula of number of copies = (amount * 6.022×10^{23}) / (length * 1×10^{9} * 650), where amount is DNA concentration, the length is the cloned DNA fragment length. The ligated plasmids of pUCm-Uni were 10-fold serially diluted from 1×10^5 to 1×10^9 copies·μL^{-1}, and C_T values for each dilution were examined by RT-qPCR (LightCycler96, Roche) with 2 x TB Green Premix (Takara) and the cycling programs of initial denaturation for 10 min at 95 °C, then 40 cycles of 10 s at 95 °C, 10 s at 63 °C and 10 s at 72 °C, and followed by the dissociation curve analysis. Then the standard curves for Uni (340-514) were constructed, and the fecal bacterial load were obtained from the ratio of Uni to feces weight (g) in the fecal DNA samples. All the tests were repeated for three times.

Text S2. Hematoxylin and eosin staining

Identical segments of liver tissues were harvested after sacrifice and rinsed with ice-cold PBS. Then, liver tissues were fixed in 4% paraformaldehyde fixative solution immediately and embedded in paraffin. The paraffin-embedded tissue sections (5 µm thick) were stained with hematoxylin and eosin (H&E) and observed under a light microscope. The degree of hepatic pathological injury was evaluated with the inflammation of the portal area and lobule and fusion necrosis, each parameter was graded on a scale of 0-4 (0: absent; 1: mild; 2: moderate; 3: severe; 4: very severe), and the score evaluation of liver sections was performed as previously described [1].

References

1. Wiersinga WJ, de Vos AF, de Beer R, et al. Inflammation patterns induced by different Burkholderia species in mice. Cell Microbiol. 2008 Jan;10(1):81-7.

II. Supplementary Figures

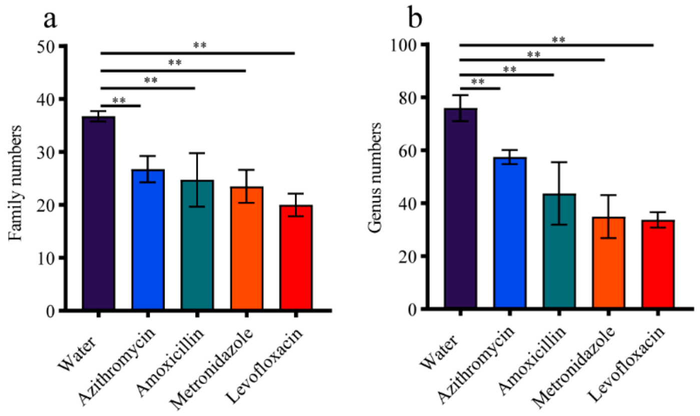

Figure S1. The statistic numbers of gut microbiota at the family and genus level.

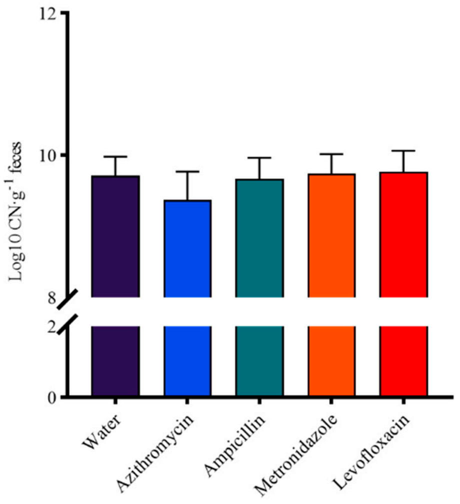

Figure S2. The total bacterial load in fecal samples quantified via RT-qPCR. CN: copy numbers of 16S rRNA gene.

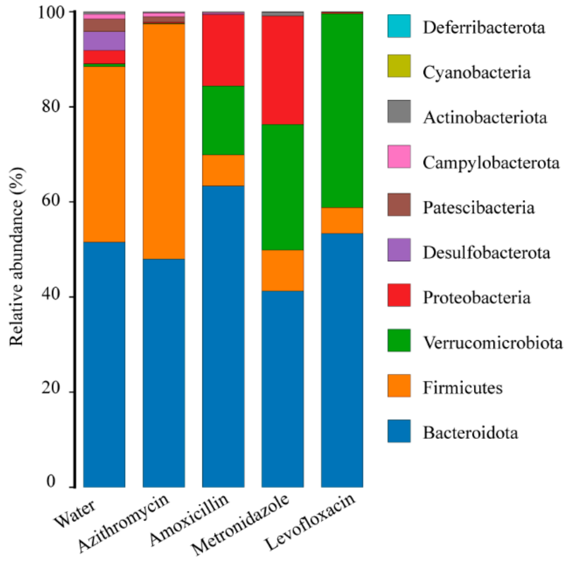

Figure S3. The relative abundance of gut microbiota at the phylum level.

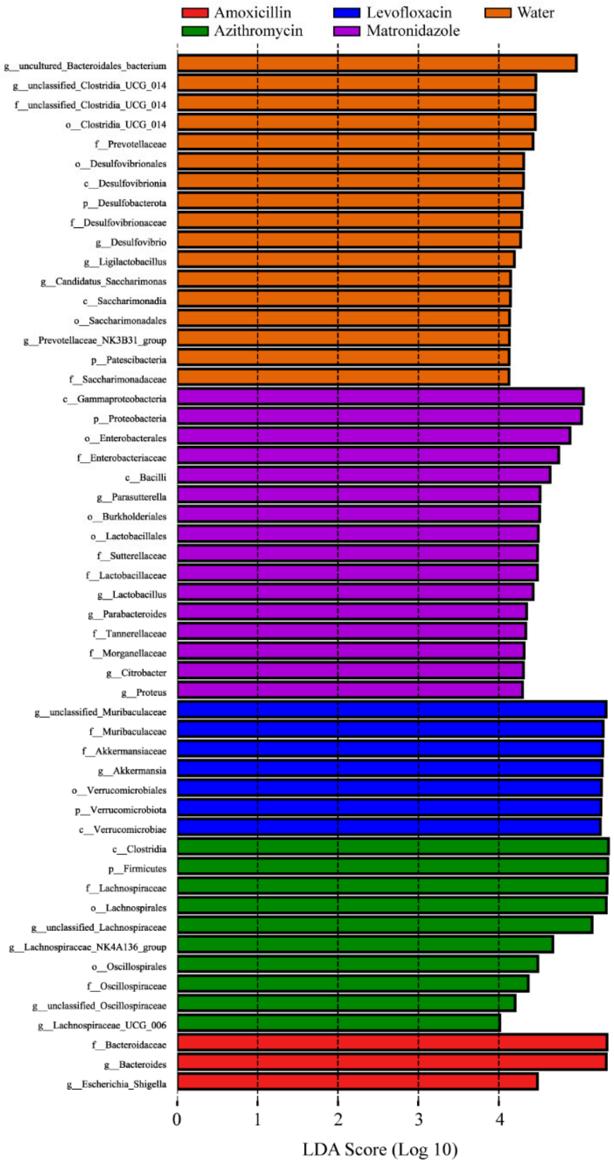

Figure S4. Linear discriminant analysis (LDA) value distribution histogram among the groups of normal, azitromycin, amoxicillin, metronidozole, and levofloxacin. LDA>4.

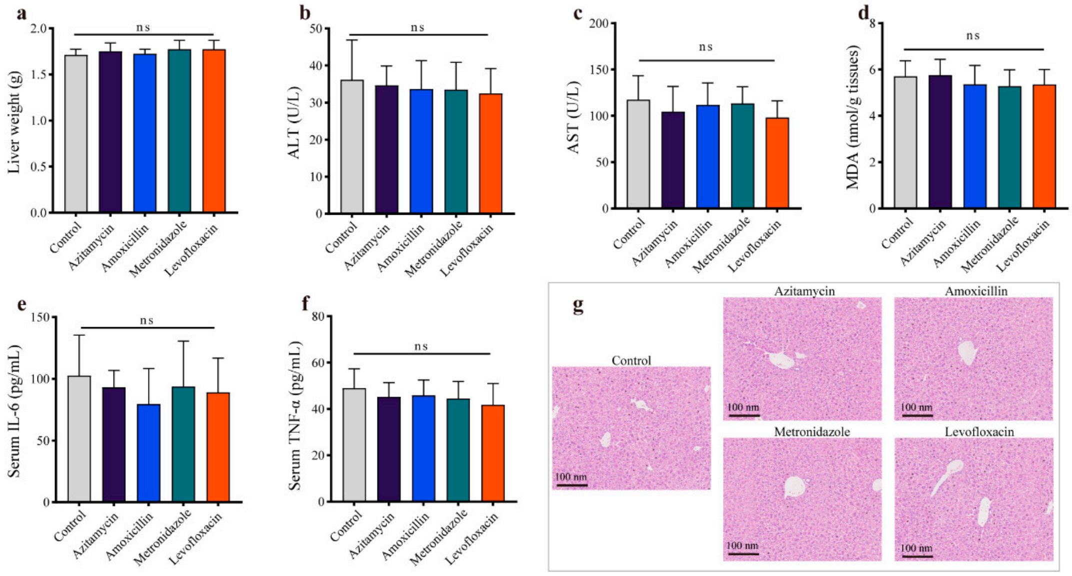

Figure S5. The signle antibiotic treatments did not induce liver injury. (a) The liver weight, the enzymatic activity of (b) ALT and (c) AST in serum, (d) the MDA level in liver tissues, the serum level of IL-6 (e) and TNF-α (f), and (g) the representative H&E staining images of liver. ns: no significant difference, ns: $p>0.05$.

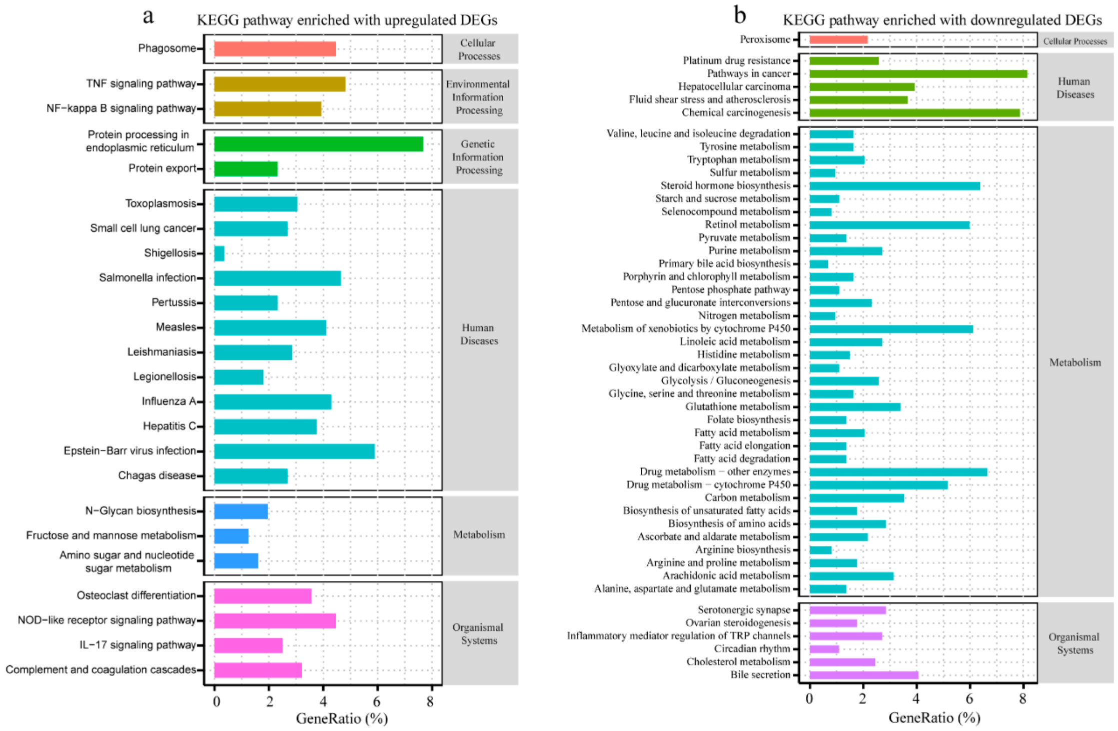

Figure S6. The KEGG pathways enriched with (a) upregulated DEGs and (b) downregulated DEGs between the groups of water and sham. The selecting criteria is false discovery rates (FDR) < 0.05, GeneRatio indicates the number of genes annotated to a pathway within the total DEGs.

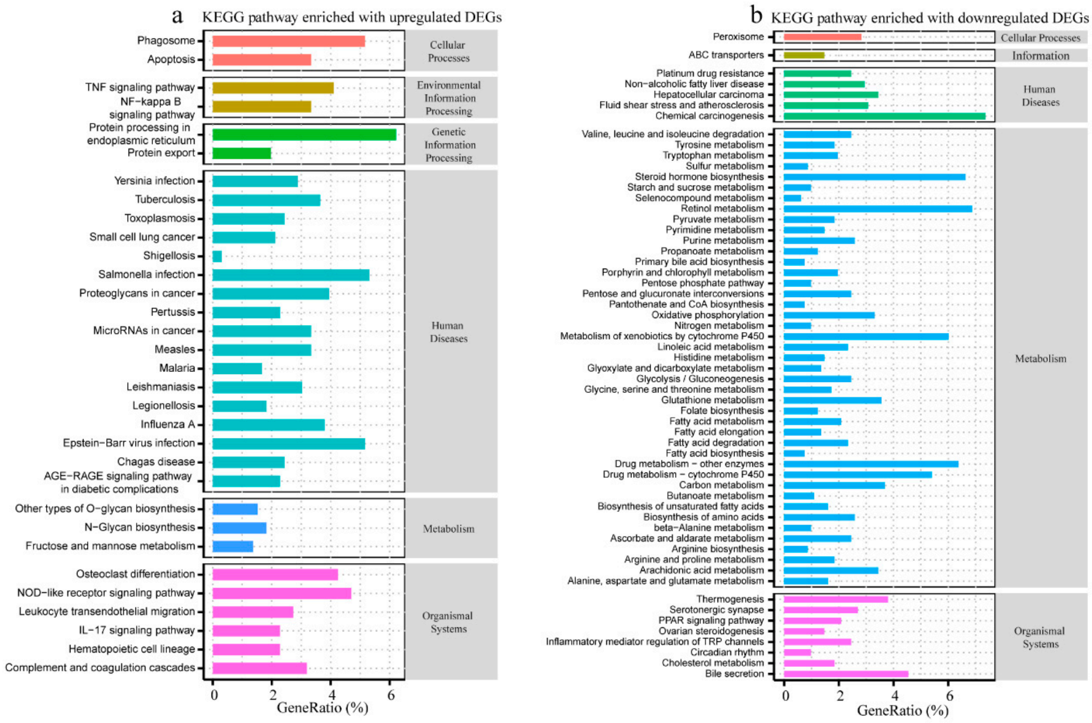

Figure S7. The KEGG pathways enriched with (a) upregulated DEGs and (b) downregulated DEGs between the groups of amoxicillin and sham. The selecting criteria is false discovery rates (FDR) < 0.05, GeneRatio indicates the number of genes annotated to a pathway within the total DEGs.

III Supplemental Tables

Table S1. Primer sequences used for gene expression analysis using RT-qPCR

Acaca-F	GTTCTGTTGGACAACGCCTTCAC
Acaca-R	GGAGTCACAGAAGCAGCCCATT
Acacb-F	AGAAGCGAGCACTGCAAGGTTG
Acacb-R	GGAAGATGGACTCCACCTGGTT
Cyp7a1-F	CACCATTCCTGCAACCTTCTGG
Cyp7a1-R	ATGGCATTCCCTCCAGAGCTGA
Cyp3a11-F	ACAGCACTGGTCAGAGCCTGAA
Cyp3a11-R	GAGAGCAAACCTCATGCCAAGG
FGF2-F	AAGCGGCTCTACTGCAAGAACG
FGF2-R	CCTTGATAGACACAACTCCTCTC
A2m-F	TCCCACAGAGACTACACGAAGC
A2m-R	GGACTGTCACTTCCATTTCAGCC
Crp-F	GATTCCTGAGGCTCCAACACAC
Crp-R	ACAGTGTAGCCCTTGTGCAGAC
SAA1-F	GGAGTCTGGGCTGCTGAGAAAA
SAA1-R	TGTCTGTTGGCTTCCTGGTCAG
Il-1b-F	CAACCAACAAGTGATATTCTCCATG
Il-1b-R	GATCCACACTCTCCAGCTGCA
Il-6-F	CACTTCACAAGTCGGAGGCT
Il-6-R	CTGCAAGTGCATCATCGTTGT
Il-17aF	GCTCCAGAAGGCCCTCAGA
Il-17aR	AGCTTTCCCTCCGCATTGA
Ccl2-F	GCTACAAGAGGATCACCAGCAG
Ccl2-R	GTCTGGACCCATTCCTTCTTGG
Ccl7-F	CAGAAGGATCACCAGTAGTCGG
Ccl7-R	ATAGCCTCCTCGACCCACTTCT
Cxcl13-F	CATAGATCGGATTCAAGTTACGCC
Cxcl13-R	GTAACCATTTGGCACGAGGATTC
Cxcl14-F	TACCCACACTGCGAGGAGAAGA
Cxcl14-R	CGCTTCTCGTTCCAGGCATTGT
18S-F	GTAACCCGTTGAACCCCATT
18S-R	CCATCCAATCGGTAGTAGCG

The supplementary data ranging from Table S2 to Table S8 were provided in separated excel files.

Table S2. The relative abundance of gut microbiota at the level of phylum, order, family, genus, and species.

Table S3. The KEGG pathways (including 3 levels) predicted by PICRUSt2 based on gut microbiota data.

Table S4. The relative abundance of Enterobacteriaceae in facultatively anaerobic

bacteria predicted by BugBase.

Table S5. The differentially expressed genes between normal CLP and sham-operated mice, the screen criteria were $|Log_2 FC| > 1$ and $FDR < 0.01$.

Table S6. The results of Gene Set Enrichment Analysis (GESA) based on the whole expressed genes of CLP (water) and sham-operated mice.

Table S7. The differentially expressed genes between amoxicillin-treated and sham-operated mice, the screen criteria were $|Log_2 FC| > 1$ and $FDR < 0.01$.

Table S8. The results of GESA based on the whole expressed genes of CLP (water) and amoxicillin-treated CLP mice.
